# Efficacy of combined orthokeratology and 0.01% atropine solution for slowing axial elongation in children with myopia: a 2-year randomised trial

**DOI:** 10.1038/s41598-020-69710-8

**Published:** 2020-07-29

**Authors:** Nozomi Kinoshita, Yasuhiro Konno, Naoki Hamada, Yoshinobu Kanda, Machiko Shimmura-Tomita, Toshikatsu Kaburaki, Akihiro Kakehashi

**Affiliations:** 1 Department of Ophthalmology, Saitama Medical Centre, Jichi Medical University, 1-847 Amanuma-cho, Omiya-ku, Saitama-shi, Saitama, 330-8503 Japan; 2 Konno Eye Clinic, Saitama, Japan; 3 Omiya Hamada Eye Clinic, Saitama, Japan; 4 Department of Hematology, Saitama Medical Centre, Jichi Medical University, Saitama, Japan

**Keywords:** Diseases, Medical research

## Abstract

Eighty Japanese children, aged 8–12 years, with a spherical equivalent refraction (SER) of − 1.00 to − 6.00 dioptres (D) were randomly allocated into two groups to receive either a combination of orthokeratology (OK) and 0.01% atropine solution (combination group) or monotherapy with OK (monotherapy group). Seventy-three subjects completed the 2-year study. Over the 2 years, axial length increased by 0.29 ± 0.20 mm (*n* = 38) and 0.40 ± 0.23 mm (*n* = 35) in the combination and monotherapy groups, respectively (*P* = 0.03). Interactions between combination treatment and age or SER did not reach significance level (age, *P* = 0.18; SER, *P* = 0.06). In the subgroup of subjects with an initial SER of − 1.00 to − 3.00 D, axial length increased by 0.30 ± 0.22 mm (*n* = 27) and 0.48 ± 0.22 mm (*n* = 23) in the combination and monotherapy groups, respectively (*P* = 0.005). In the − 3.01 to − 6.00 D subgroup, axial length increased by 0.27 ± 0.15 mm (*n* = 11) and 0.25 ± 0.17 mm (*n* = 12) in the combination and monotherapy groups, respectively (*P* = 0.74). The combination therapy may be effective for slowing axial elongation, especially in children with low initial myopia.

## Introduction

The prevalence of myopia is increasing worldwide^1–3^ and younger generations are affected more than others^4–6^. Myopia progression in children is strongly associated with axial elongation^7^. Retinal changes caused by axial elongation of high myopia increase the risks of myopic maculopathy, retinal detachment, glaucoma, and resulting blindness^4,8,9^. Although controlling axial elongation is vital for reducing the risk of these complications, no treatment has yet been established to halt axial elongation.

Recent studies, however, have provided evidence of effective methods to slow the progression of myopia. The Atropine for the Treatment of Myopia (ATOM) 1 study demonstrated that treatment with 1% atropine ophthalmic solution significantly suppressed the progression of myopia by about 80% compared to placebo over a 2-year period^10^. However, 1% atropine produced secondary unwanted effects such as pupil dilation and loss of accommodation^11^, and rebound effect after cessation of treatment^12^. Thus, in the ATOM2 study, 0.5%, 0.1%, and 0.01% atropine ophthalmic solutions were examined, leading to reduced myopia progression by about 75%, 70%, and 60%, respectively, compared to placebo in the ATOM1 study over a 2-year period^13–15^. Because the secondary unwanted effects and rebound effect were scarcely seen in the 0.01% atropine group compared to the higher concentrations of atropine, 0.01% atropine was the most recommended. However, axial elongation was not suppressed significantly in the ATOM2 study. Recently, the low-concentration atropine for myopia progression (LAMP) study compared the efficacy of 0.05%, 0.025%, and 0.01% atropine ophthalmic solutions and placebo for suppressing myopia progression and axial elongation^16,17^. The results showed that 0.05% atropine was the most effective for suppressing of myopia progression and axial elongation with tolerable secondary unwanted effects. However, since atropine ophthalmic solutions cannot provide myopia correction, visual correction with spectacles, contact lenses, or orthokeratology (OK) lenses is needed.

OK uses specially designed hard contact lenses that flatten the central corneal curve to reduce myopia when worn at night. This procedure can achieve full correction of myopia without spectacles or contact lenses during the day. Recently, several reports from various countries have described the efficacy of OK in slowing axial elongation in children^18–21^. A meta-analysis of OK reported that axial elongation was reduced by an average of 43% compared to single vision spectacles over a 2-year period^22^. Moreover, a meta-analysis of 16 interventions for myopia control in children by Huang et al. showed that the suppressive effect of OK was smaller than that of high- and moderate-dose atropine ophthalmic solutions and was equivalent to that of low-dose atropine ophthalmic solution^23^. However, unlike atropine ophthalmic solutions, OK has the merit of correcting myopia, thereby enabling freedom from spectacles or contact lenses during the day.

The anti-myopia mechanisms of atropine and OK remain unclear. Those of atropine have been suggested to involve the blocking of muscarinic receptors and/or the stimulating of α2-adrenoceptors involved in axial elongation in the retina and sclera^24–27^. In contrast, those of OK have been theorized to involve the reduction of peripheral hyperopic defocus^28–30^ and/or the increase in higher order aberration, especially coma-like aberration^31^, through a redistribution of the corneal epithelium in which the central cornea becomes thinner and the mid-periphery becomes thicker^32^. Generally, atropine is considered to slow the progression of myopia by a pharmaceutical mechanism and OK by an optical mechanism. Combining treatments with different mechanisms of action may be more effective than monotherapy in slowing the progression of myopia. Although 1% atropine is not usable in clinical practice due to secondary unwanted effects and treatment cessation rebound, using 0.01% atropine together with OK might be a usable and optimal treatment option. We thus conducted a prospective interventional, parallel-group randomised clinical trial of combination treatment with OK and 0.01% atropine ophthalmic solution to investigate their additive or synergistic effects for slowing axial elongation in children with myopia. We reported the first-year results of this study in 2018^33^. Since then, although similar preliminary studies have been reported^34–36^, the results of the 2-year prospective study have not yet been reported. In this report, we describe the results at the 2-year follow-up of the prospective, randomised clinical trial.

## Methods

The design of this clinical trial registered on the UMIN-CTR (Registration No: UMIN000014362; Date of Registration: June 24, 2014) was described previously^33^. The methods are summarised below.

### Participants

Japanese boys and girls, aged 8–12 years, who elected to undergo OK treatment at Konno Eye Clinic or Omiya Hamada Eye Clinic were included. The inclusion criteria were as follows: a cycloplegic spherical equivalent refraction (SER) of − 1.00 to − 6.00 dioptres (D) in both eyes; astigmatism of ≤ 1.50 D in both eyes; anisometropia of ≤ 1.50 D; and a best-corrected visual acuity of ≤ 0.00 logarithm of the minimum angle of resolution (logMAR) unit in each eye. The exclusion criteria were as follows: the presence of ocular disorders such as strabismus and amblyopia; systemic disorders such as cardiac or respiratory illness; low birth weight of ≤ 1,500 g; a history of hypersensitivity to atropine; and using OK and/or atropine ophthalmic solutions.

### Baseline and group allocation

Since the central corneal thickness stabilises after the first 1–2 months of OK therapy^32,37,38^, the axial length measurement at month 3 of OK therapy was used as the baseline value, based on studies conducted by Kakita et al. and Hiraoka et al.^20,21^ We considered that the OK lenses fit well based on the corneal topography images of a typical concentric bull's eye with an uncorrected distance visual acuity of ≤ 0.00 logMAR unit that was obtained in the daytime in each eye. At baseline, participants who had successfully been wearing the OK lenses for 3 months after study entry were randomly allocated by a third person into two groups to receive either a combination of OK and 0.01% atropine ophthalmic solution (combination group) or monotherapy with OK (monotherapy group). Subjects in the combination group started using 0.01% atropine in both eyes once nightly at 3 months after beginning the OK treatment. We used a stratified randomisation method to control for age and SER at enrolment. Specifically, children were divided into four blocks by age (8.0–10.9 years and 11.0–12.9 years) and SER (− 1.00 to − 3.00 D and − 3.01 to − 6.00 D), and the children in each block were randomly allocated into the two groups.

### Orthokeratology lenses

The OK lenses used in this study were four-zone reverse geometry lenses (Breath-O Correct; Universal View Co., Ltd., Tokyo, Japan), as previously described^33^. Subjects in both groups were instructed to wear their OK lenses on both eyes every night for at least 6 consecutive hours.

### Atropine ophthalmic solution

The 0.01% atropine ophthalmic solution for the combination group was specially prepared for this study by Fuji Yakuhin Co., Ltd. (Saitama, Japan) by diluting Nitten Atropine Ophthalmic Solution 1% (Nihon Pharmaceutical Co., Ltd., Nagoya, Japan) with physiologic saline at a ratio of 1:99 in a sterile manner. Because of the physical, chemical, and microbiological stability of 0.01% atropine solution conserved inside polyethylene containers for six months at 25 °C or 5 °C^39^, the 0.01% atropine solution was stored in a 5 ml polypropylene container in cold conditions and the shelf life for an unopened product was 3 months. Since the benzalkonium chloride preservative was also diluted, the solution was used within a week after opening except in cases where the tip of the container was contaminated. Subjects in the combination group were instructed to instil the 0.01% atropine into both eyes without their eyelids or eyelashes touching the tip of the container, once daily at night, at least 5 min before inserting the OK lenses.

### Measurements

Subjects in both groups visited each clinic every 3 months for measurements of the axial length, corneal endothelial cell density, intraocular pressure (IOP), uncorrected distant and near visual acuities, refraction and corneal topography. The compliance to the use of OK lenses and 0.01% atropine ophthalmic solution was also evaluated using interview sheets at each visit. Specifically, children and parents or guardians received interview sheets questioning their average weekly use of OK lenses and 0.01% atropine ophthalmic solution since their last visit. When the uncorrected distant visual acuity changed by more than 0.30 logMAR unit due to progression of myopia during the follow-up period, the OK lenses were re-prescribed. At that time, we confirmed by corneal topography whether the decrease in visual acuity was due to decentration of the OK lenses or progression of myopia. Subjects underwent a slit-lamp examination to assess the fitting of the OK lenses and the presence or absence of adverse events. Subjects were withdrawn from the study if any adverse events occurred that would not allow further use of either the OK lenses or the 0.01% atropine ophthalmic solution. Axial length was measured using an IOLMaster (Carl Zeiss, Dublin, CA) by examiners blinded to the refractive status at enrolment and the group allocation of participants. The corneal endothelial cell density was measured using a Noncon ROBO CA SP-8800 (Konan Medical, Inc., Tokyo, Japan). The IOP and refraction were measured using a TONOREF II (NIDEK CO., LTD., Aichi, Japan). The corneal topography was measured using a TMS-4 (Tomey Corporation, Aichi, Japan). All measurements were non-contact. To obtain a mean value for analyses, the axial length was measured five or more times and the IOP and refraction were measured three or more times. All participants paid a discounted fee for the treatments delivered in the context of this study.

### Statistical analyses

Based on the findings from previously published studies^15,18–21^, it was estimated that increases in axial length over a 2-year period would be 0.28 mm in the combination group and 0.45 mm in the monotherapy group, with a standard deviation of 0.25 mm. To detect this difference as an α error of 0.05 and a β error of 0.20, a sample size of 68 subjects (34:34) was required. Assuming that 15% of subjects would drop out after allocation into two groups, a sample size of 80 subjects (40:40) was required at baseline. As a primary endpoint of this study, changes in axial length over 2 years (from baseline to the follow-up examination at 24-month) were compared between the combination and monotherapy groups using an unpaired *t*-test after confirming that the data were normally distributed and that the variances were equal in the two groups. As post-hoc exploratory analyses, changes in axial length from enrolment to baseline and from baseline to the follow-up examinations at 6-, 12-, and 18-months were compared between the two groups using the unpaired *t*-test, after repeated-measures ANOVA for four time points (over 6-, 12-, 18-, and 24-month periods) was performed. The relationships between the change in axial length over 2 years and the age and SER at enrolment were analysed using a Pearson’s correlation coefficient and linear regression analysis. The interactions between combination treatment and age and between combination treatment and SER were analysed using a multiple linear regression analysis for changes in axial length over 1 year or 2 years. Changes in axial length over 6-, 12-, 18-, and 24-month periods stratified by age and SER at enrolment were compared between the two groups using an unpaired *t*-test. The effects of adding 0.01% atropine ophthalmic solution to the OK therapy were assessed by comparing changes in corneal endothelial cell density, IOP, and uncorrected distant and near visual acuities between the two groups at the end of the 2-year period using an unpaired *t*-test. Subjects with compliance rates of < 60% (corresponding to 4.2 days per week) to the treatment protocol of OK lenses and/or 0.01% atropine ophthalmic solution during the 2-year period were excluded from statistical analyses. All measurements obtained from both eyes of the same subject were averaged for statistical analyses. All statistical analyses were performed using the statistical software EZR (Saitama Medical Centre, Jichi Medical University, Saitama, Japan)^40^. The data are expressed as the mean ± standard deviation. Differences were considered statistically significant for *P* values < 0.05.

### Informed consent and ethics

The parents or legal guardians of each child provided written informed consent after they received a full explanation of the expected benefits and potential risks of treatment. The participating children also provided informed assent after they reviewed an age-appropriate document. This study was conducted according to the tenets of the Declaration of Helsinki; the ethics committee at Saitama Medical Centre, Jichi Medical University approved the study protocol.

## Results

### Subjects and their characteristics

**Figure 1:**
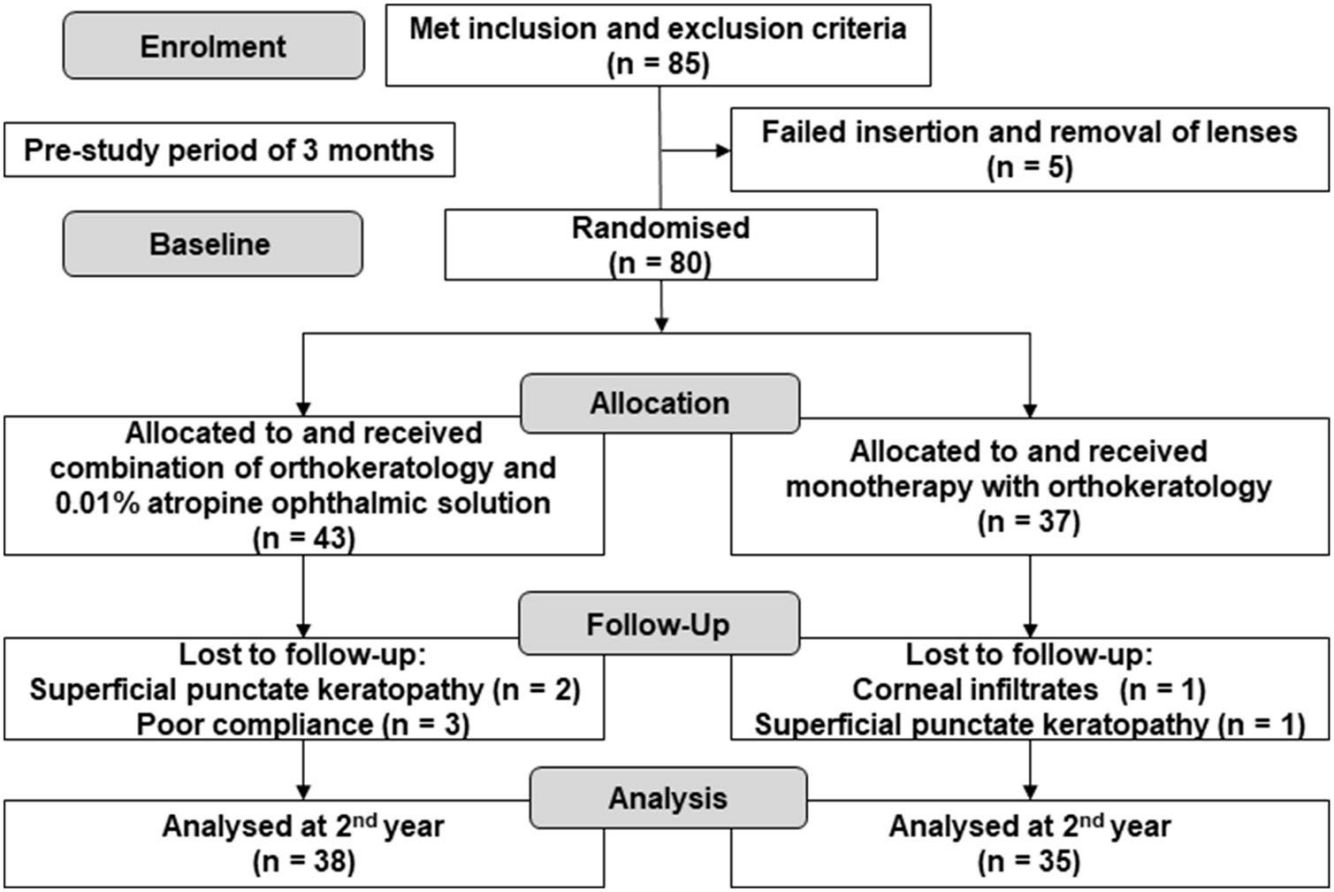
Flowchart representing the process undergone by study participants.

A total of 85 children who met the inclusion and exclusion criteria in this study were recruited to undergo fitting with the OK lenses between June 30, 2014 and December 31, 2016. Five children failed insertion and removal of the lenses before they were admitted to the study. A total of 80 subjects who had successfully been wearing OK lenses for 3 months after entering the study were randomly allocated into the two groups (43 in the combination group and 37 in the monotherapy group). By March 31, 2019, a total of 73 subjects (38 in the combination group and 35 in the monotherapy group) had completed the 2-year study. One subject in the monotherapy group dropped out because of infiltrates in the upper cornea of the right eye. Two subjects in the combination group and one subject in the monotherapy group dropped out because of worsening superficial punctate keratopathy (SPK) in both eyes. In the combination group, two subjects were excluded because of compliance rates of < 60% to the treatment protocol of OK lenses and 0.01% atropine ophthalmic solution and one subject was excluded because of a compliance rate of < 60% to the treatment protocol of 0.01% atropine solution only. No subjects in the monotherapy group were excluded because of compliance rates of < 60% to the treatment protocol of OK lenses (Fig. 1). Table 1 outlines the characteristics at enrolment of subjects who completed the 2-year study in both the combination and monotherapy groups. The age, sex, SER, axial length, corneal endothelial cell density, IOP, and uncorrected distant visual acuity were balanced between the two groups, with no statistical differences between them.

**Table 1:** Characteristics at enrolment of subjects who completed the 2-year study in the orthokeratology and 0.01% atropine combination group and the orthokeratology monotherapy group.

	Combination group (*n* = 38)	Monotherapy group (*n* = 35)	*P* value
Age (years)	10.33 ± 1.59	10.37 ± 1.65	0.91*
Sex (male:female)	18:20	18:17	0.74^†^
Spherical equivalent refraction (D)	− 2.60 ± 1.29	− 2.72 ± 1.31	0.69*
Axial length (mm)	24.69 ± 0.58	24.86 ± 0.81	0.33*
Corneal endothelial cell density (/mm^2^)	3,338 ± 241	3,238 ± 225	0.07*
Intraocular pressure (mmHg)	14.2 ± 2.3	14.3 ± 2.3	0.83*
Uncorrected distant visual acuity (logMAR)	0.76 ± 0.25	0.78 ± 0.28	0.67*

Unless indicated otherwise, the data are expressed as the mean ± standard deviation.

*D* dioptres, *logMAR* logarithm of the minimum angle of resolution.

*Unpaired *t*-test.

^†^Mann–Whitney *U* test.

### Changes in measurements

Changes in axial length, corneal endothelial cell density, IOP, and uncorrected distant and near visual acuities over 2 years were compared between the two groups (Table 2). Planned statistical analysis for the primary endpoint showed that the axial length over 2 years increased by 0.29 ± 0.20 mm in the combination group and 0.40 ± 0.23 mm in the monotherapy group (*P* = 0.03, unpaired *t*-test). The combination of OK and 0.01% atropine was 28% more effective in slowing axial elongation than OK monotherapy. No significant differences between the combination and monotherapy groups were observed in terms of corneal endothelial cell density, IOP, or uncorrected distant and near visual acuities. The results of the post-hoc exploratory analyses are shown below.

**Table 2:** Changes in axial length, corneal endothelial cell density, intraocular pressure, and uncorrected distant and near visual acuities over 2 years in the orthokeratology and 0.01% atropine combination group and the orthokeratology monotherapy group.

Parameter	Combination group (*n* = 38)	Monotherapy group (*n* = 35)	*P* value*
Axial length (mm)	0.29 ± 0.20	0.40 ± 0.23	0.03
Corneal endothelial cell density (/mm^2^)	− 77 ± 198	− 64 ± 243	0.79
Intraocular pressure (mmHg)	− 1.1 ± 2.2	− 0.6 ± 2.1	0.28
Uncorrected distant visual acuity (logMAR)	0.07 ± 0.13	0.08 ± 0.10	0.64
Uncorrected near visual acuity (logMAR)	0.01 ± 0.09	0.03 ± 0.07	0.51

Data are expressed as the mean ± standard deviation.

*logMAR* logarithm of the minimum angle of resolution.

*Unpaired *t*-test.

### Time courses of changes in axial length

**Figure 2:**
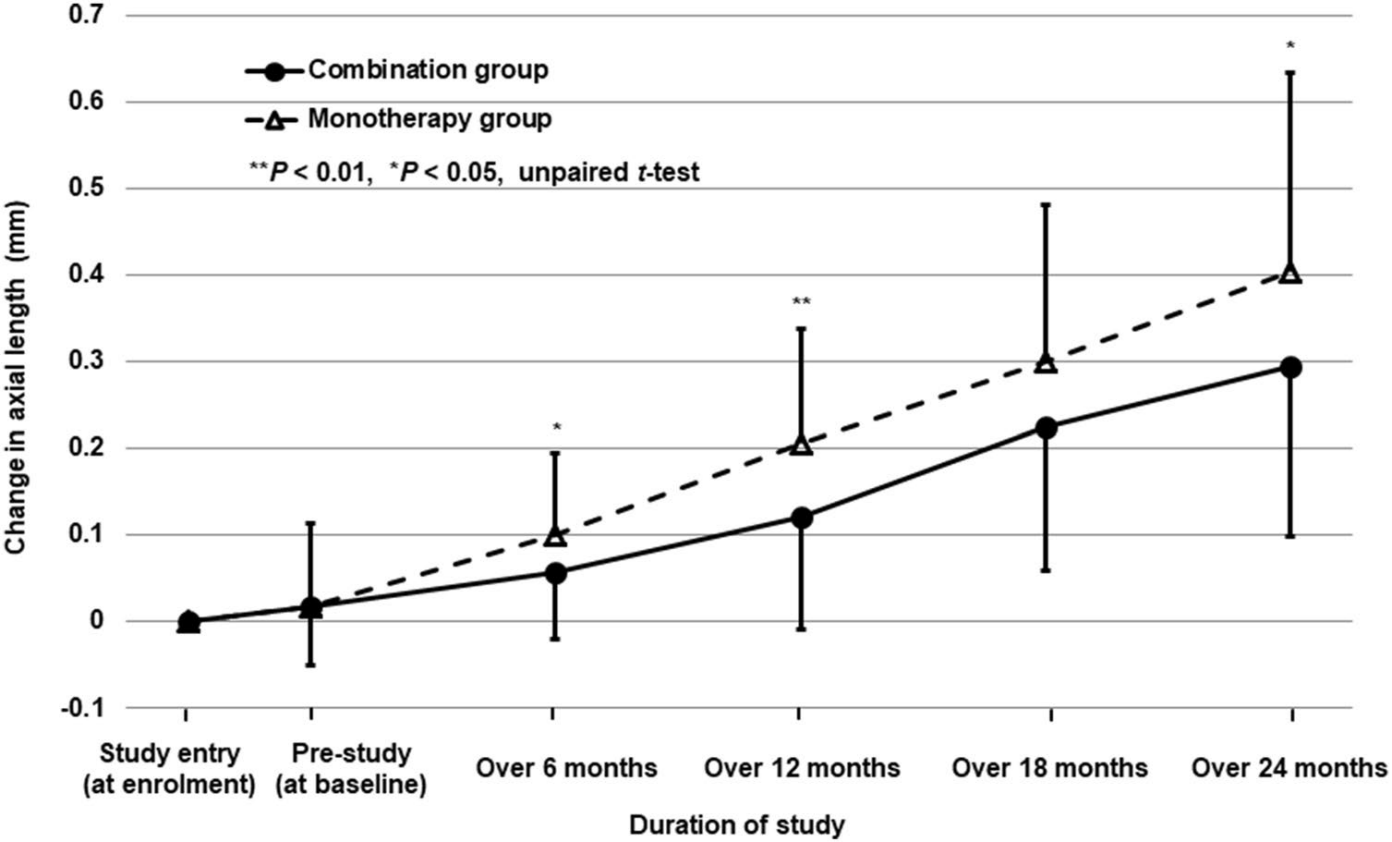
Time courses of changes in axial length in the orthokeratology and 0.01% atropine combination group and the orthokeratology monotherapy group. Error bars represent the standard deviation.

Figure 2 shows the time courses of changes in axial length in the combination and monotherapy groups. Repeated-measures ANOVA for four time points (over 6-, 12-, 18-, and 24-month periods) differed significantly between the combination and monotherapy groups (*P* = 0.02). During the 3-month pre-study period, changes in axial length did not differ significantly between the combination group (0.02 ± 0.07 mm) and the monotherapy group (0.02 ± 0.10 mm) (*P* = 0.95, unpaired *t*-test). The changes in axial length over 6 months and 12 months (i.e., in the first year) were significantly smaller in the combination group (0.06 ± 0.08 mm and 0.12 ± 0.08 mm) than in the monotherapy group (0.10 ± 0.09 mm and 0.21 ± 0.13 mm) (*P* = 0.03 and 0.008, unpaired *t*-test), although those over 18 months and from 12 to 24 months (i.e., in the second year) did not differ significantly between the combination group (0.23 ± 0.17 mm and 0.17 ± 0.10 mm) and the monotherapy group (0.30 ± 0.18 mm and 0.20 ± 0.13 mm) (*P* = 0.07 and 0.36, unpaired *t*-test).

### Changes in axial length according to age and SER

**Figure 3:**
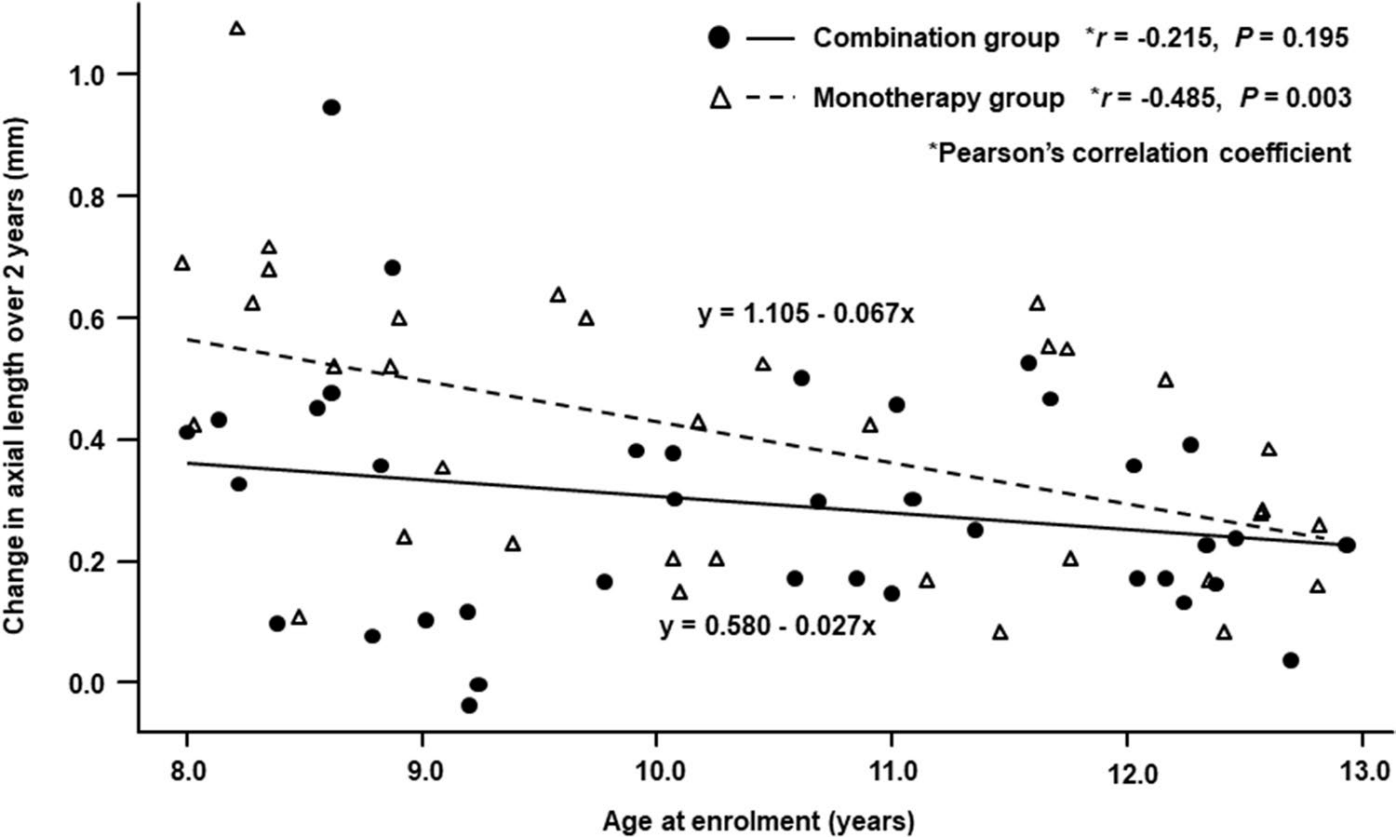
Scatter plots of the change in axial length over 2 years versus age at enrolment in the orthokeratology and 0.01% atropine combination group and the orthokeratology monotherapy group.

**Figure 4:**
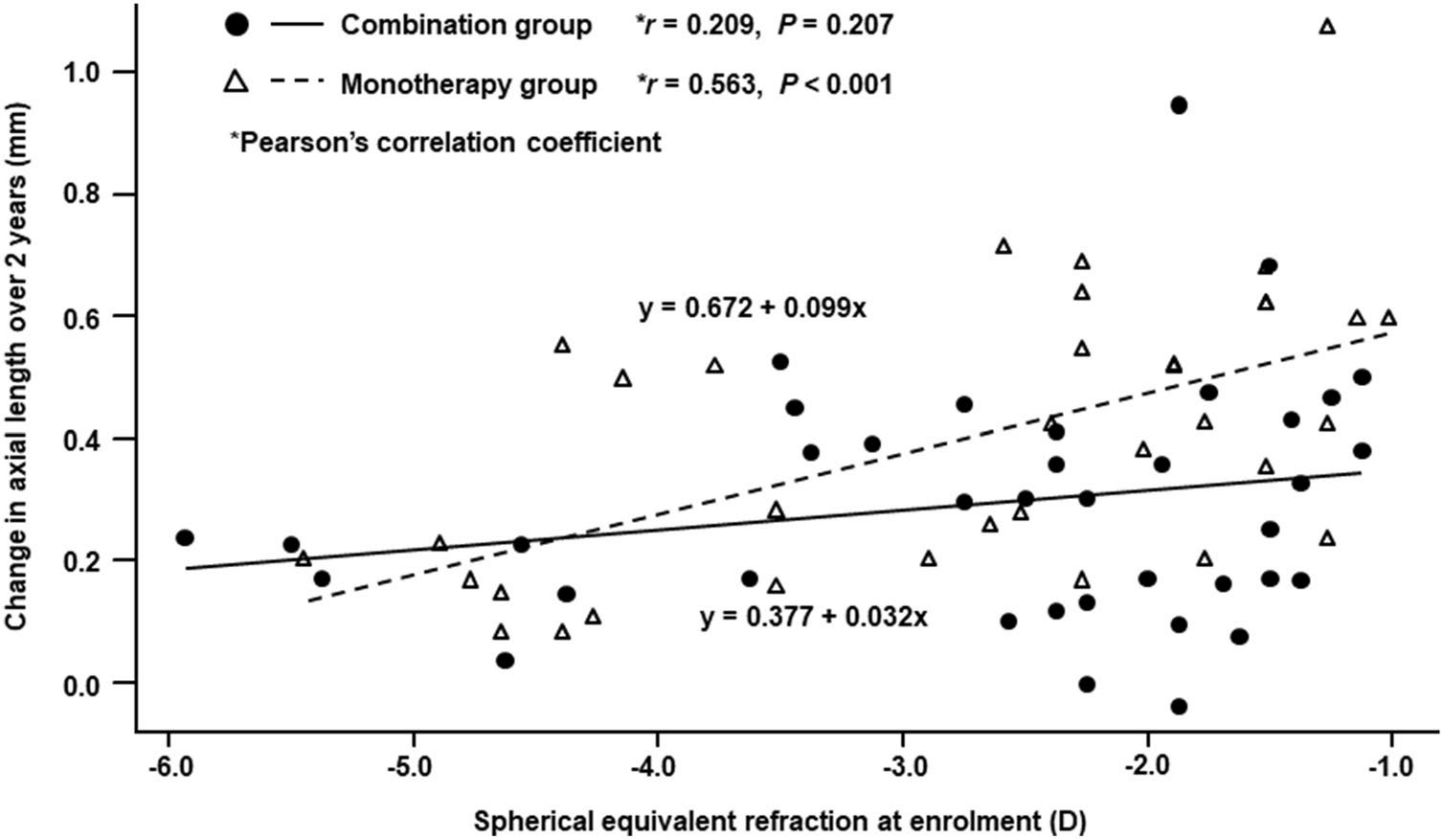
Scatter plots of the change in axial length over 2 years versus spherical equivalent refraction at enrolment in the orthokeratology and 0.01% atropine combination group and the orthokeratology monotherapy group. *D*  dioptres.

Figure 3 shows the scatter plots of the changes in axial length over 2 years versus age at enrolment in the two groups. A significant negative correlation was observed between the change in axial length and age in the monotherapy group (Pearson’s correlation coefficient; *r* =  −0.485, *P* = 0.003), in which the axial length increased more in younger subjects. In contrast, no significant correlation was observed between the two parameters in the combination group (Pearson’s correlation coefficient; *r* =  −0.215, *P* = 0.20). Figure 4 shows the scatter plots of the changes in axial length over 2 years versus SER at enrolment in the two groups. A significant positive correlation was observed between the change in axial length and SER in the monotherapy group (Pearson’s correlation coefficient; *r* = 0.563, *P* < 0.001), in which the axial length increased more in subjects with lower initial myopia. In contrast, no significant correlation was observed between the two parameters in the combination group (*r* = 0.209, *P* = 0.21). When the interactions between combination treatment and age and between combination treatment and SER for changes in axial length over 1 year or 2 years were analysed by multiple linear regression analysis, a tendency toward positive interactions was observed between combination treatment and SER (1 year: *P* = 0.02, 2 years: *P* = 0.06), whereas interactions between combination treatment and age were not significant (1 year: *P* = 0.35, 2 years: *P* = 0.18).

### Stratification by SER

**Figure 5:**
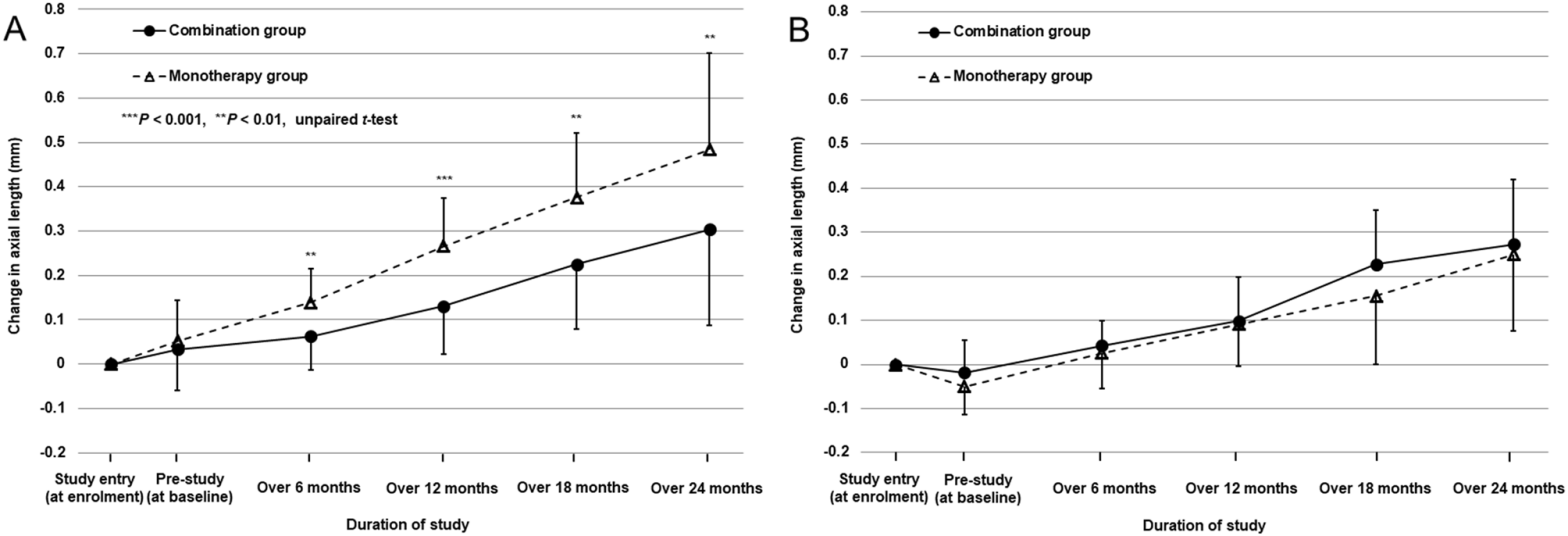
Time courses of changes in axial length in the orthokeratology and 0.01% atropine combination group and the orthokeratology monotherapy group stratified by spherical equivalent refraction (SER) at enrolment. **A**: subjects with a SER of − 1.00 to − 3.00 dioptres (D), **B**: subjects with a SER of − 3.01 to − 6.00 D. Error bars represent the standard deviation.

We stratified the subjects by SER at enrolment (− 1.00 to − 3.00 D vs. − 3.01 to − 6.00 D) and compared the changes in axial length between the combination and monotherapy groups (Fig. 5A, B). In the subgroup of subjects with a SER of − 1.00 to − 3.00 D (27 subjects in the combination group and 23 in the monotherapy group), all changes in axial length over 6-, 12-, 18-, and 24-month periods were significantly smaller in the combination group (0.06 ± 0.08 mm, 0.13 ± 0.14 mm, 0.23 ± 0.18 mm, and 0.30 ± 0.22 mm, respectively) than in the monotherapy group (0.14 ± 0.08 mm, 0.27 ± 0.11 mm, 0.38 ± 0.15 mm, and 0.48 ± 0.22 mm, respectively) (*P* = 0.001, 0.0005, 0.003, and 0.005, respectively, unpaired *t*-test). The ages at enrolment in this subgroup did not differ significantly between the combination group (9.95 ± 1.40 years) and the monotherapy group (10.06 ± 1.67 yeas) (*P* = 0.79, unpaired *t*-test). During the 2-year period, the combination of OK and 0.01% atropine was 38% more effective in slowing axial elongation than OK monotherapy, yielding a larger difference in this subgroup compared to all subjects. In contrast, in the subgroup of subjects with a SER of − 3.01 to − 6.00 D (11 subjects in the combination group and 12 in the monotherapy group), all changes in axial length over 6-, 12-, 18-, and 24-month periods did not differ significantly between the combination group (0.04 ± 0.06 mm, 0.10 ± 0.10 mm, 0.23 ± 0.12 mm, and 0.27 ± 0.15 mm, respectively) and the monotherapy group (0.03 ± 0.08 mm, 0.09 ± 0.10 mm, 0.16 ± 0.16 mm, and 0.25 ± 0.17 mm, respectively) (*P* = 0.59, 0.88, 0.24, and 0.74, respectively, unpaired *t*-test), and the ages at enrolment did not differ significantly between the combination group (11.53 ± 1.32 years) and the monotherapy group (10.96 ± 1.50 years) (*P* = 0.34, unpaired *t*-test).

### Stratification by age

We also stratified the subjects by age at enrolment (8.0–10.9 years vs. 11.0–12.9 years) and compared the changes in axial length between the combination and monotherapy groups. In the subgroup of subjects aged 8.0–10.9 years (22 subjects in the combination group and 21 in the monotherapy group), although only changes in axial length over 12- and 24-month periods were significantly smaller in the combination group (0.14 ± 0.16 mm and 0.31 ± 0.23 mm, respectively) than in the monotherapy group (0.25 ± 0.14 mm and 0.47 ± 0.23 mm, respectively) (*P* = 0.03 and 0.03, respectively, unpaired *t*-test), the changes in axial length over 6- and 18-month periods did not differ significantly between the combination group (0.07 ± 0.09 mm and 0.24 ± 0.20 mm, respectively) and the monotherapy group (0.12 ± 0.10 mm and 0.35 ± 0.18 mm, respectively) (*P* = 0.11 and 0.06, respectively, unpaired *t*-test). The SERs at enrolment in this subgroup did not differ significantly between the combination group (− 2.12 ± 0.73 D) and the monotherapy group (− 2.38 ± 1.36 D) (*P* = 0.43, unpaired *t*-test). In contrast, in the subgroup of subjects with aged 11.0–12.9 years (16 subjects in the combination group and 14 in the monotherapy group), all changes in axial length over 6-, 12-, 18-, and 24-month periods did not differ significantly between the combination group (0.03 ± 0.04 mm, 0.10 ± 0.08 mm, 0.21 ± 0.10 mm, and 0.27 ± 0.14 mm, respectively) and the monotherapy group (0.07 ± 0.09 mm, 0.15 ± 0.09 mm, 0.22 ± 0.16 mm, and 0.30 ± 0.18 mm, respectively) (*P* = 0.12, 0.11, 0.76, and 0.57, respectively, unpaired *t*-test), and the SERs at enrolment did not differ significantly between the combination group (−  3.26 ± 1.60 D) and the monotherapy group (−  3.23 ± 1.08 D) (*P* = 0.34, unpaired *t*-test). Based on the above results, the SER was judged to have a greater effect than the age.

### Adverse events

One subject in the monotherapy group developed infiltrates in the upper cornea of the right eye after 9 months. She was withdrawn from the study and switched from using OK lenses to spectacles. The corneal infiltrates resolved after topical antimicrobial therapy. Two subjects in the combination group and one subject in the monotherapy group had mild SPK in both eyes but initially remained in the study because they had no subjective symptoms. However, after their SPK worsened and subjective symptoms appeared, they were withdrawn from the study and switched from using OK lenses to spectacles. Their SPK resolved about 1 month after discontinuation of the OK lenses. No subjects in the combination group dropped out because of photophobia, impairment of near visual acuity, allergic reaction, microbial infection, or systemic adverse effects caused by the 0.01% atropine ophthalmic solution. During the study period, no subjects in either group were re-prescribed the OK lenses because their uncorrected distant visual acuities did not change by more than 0.30 logMAR unit due to progression of their myopia.

## Discussion

To our knowledge, this is the first prospective study to evaluate the efficacy of combined OK and 0.01% atropine therapy for slowing axial elongation in children with myopia over a 2-year period. As a primary endpoint of this study, axial elongation over a 2-year period was more significantly suppressed by combined OK and 0.01% atropine therapy than OK alone. Post-hoc exploratory analyses also found that repeated-measures ANOVA for four time points (over 6-, 12-, 18-, and 24-month periods) differ significantly between the two group, and axial elongation over 6- and 12- periods was more significantly suppressed by combined OK and 0.01% atropine therapy than OK alone (Fig. 2).

Table 3 shows the comparisons of axial elongation over 2 years in our study and in previous studies performed by other investigators. Chia et al. reported that the axial length in children receiving 0.5%, 0.1%, and 0.01% atropine increased by 0.27 mm, 0.28 mm, and 0.41 mm, respectively^15^. Yam et al. reported that the axial length in children receiving 0.05%, 0.025%, and 0.01% atropine increased by 0.39 mm, 0.50 mm, and 0.59 mm, respectively^16^. The axial length increase of 0.29 mm in the combination therapy group in our study was smaller than in the children receiving 0.05% atropine in the study by Yam et al. In the studies by Cho et al., Santodomingo-Rubido et al., and Hiraoka et al., which all recorded axial length measurements using the IOLMaster, the axial length increased by 0.36–0.47 mm in children receiving OK therapy and 0.63–0.71 mm in those using single vision spectacles^18–20^. In our study, the axial length increase of 0.40 mm in the OK monotherapy group was relatively consistent with the findings of these previous studies, and the combination of OK and 0.01% atropine was 28% more effective in slowing axial elongation than OK monotherapy. We acknowledge, however, that simple comparisons with these studies and ours may not be appropriate because of differences in methodologies and subjects’ ethnicities, ages, and initial SERs.

**Table 3:** Comparisons of axial elongation over 2 years in our study and previous studies performed by other investigators.

References	Ethnicity	Age range (years)	Initial SER (D)	Interventions	Change in axial length (mm)
Chia et al.^15^	Chinese	6–12	− 4.5 ± 1.5	0.5% atropine	0.27 ± 0.25
			− 4.8 ± 1.5	0.1% atropine	0.28 ± 0.27
			− 4.7 ± 1.8	0.01% atropine	0.41 ± 0.32
Yam et al.^16^	Chinese	4–12	− 3.93 ± 1.63	0.05% atropine	0.39 ± 0.35
			− 3.88 ± 1.83	0.025% atropine	0.50 ± 0.33
			− 3.99 ± 1.94	0.01% atropine	0.59 ± 0.38
Cho et al.^18^	Chinese	7–10	− 2.16 ± 0.77	OK	0.36 ± 0.24
			− 2.36 ± 0.86	Single vision spectacles	0.63 ± 0.26
Santodomingo-Rubido et al.^19^	White European	6–12	− 2.20 ± 1.09 (Sphere)	OK	0.47
			− 2.35 ± 1.17 (Sphere)	Single vision spectacles	0.69
Hiraoka et al.^20^	Japanese	8–12	− 1.89 ± 0.82	OK	0.45 ± 0.21
			− 1.83 ± 1.06	Single vision spectacles	0.71 ± 0.40
Present study	Japanese	8–12	− 2.60 ± 1.29	OK + 0.01% atropine	0.29 ± 0.20
			− 2.72 ± 1.31	OK	0.40 ± 0.23

Data are expressed as the mean ± standard deviation except for the increase in axial length of Santodomingo-Rubido et al.^19^ that are expressed as the mean.

*SER* spherical equivalent refraction, *D* dioptres, *OK* orthokeratology.

In this study, one subject in the OK monotherapy group dropped out because of corneal infiltrates, and two subjects in the combination therapy group and one subject in the OK monotherapy group dropped out because of worsening SPK. There was no difference in the numbers of patients who dropped out due to adverse events between the two groups, but it may be necessary to confirm the safety of the combination treatment in larger studies. Although we used the 0.01% atropine ophthalmic solution prepared by diluting the existing 1% atropine ophthalmic solution in a sterile manner, a preservative-free and single-use ophthalmic solution would be more preferable for combined OK and 0.01% atropine therapy to prevent corneal infection and worsening SPK. In the combination therapy group, two subjects showed poor compliance with the use of OK lenses and 0.01% atropine ophthalmic solution, and one subject showed poor compliance with the use of 0.01% atropine ophthalmic solution only. In contrast, no subjects in the OK monotherapy group showed poor compliance with the use of OK lenses. This finding might have resulted from the additional complexity of performing two different procedures each night, although self-reporting by children and their parents or guardians as a method to monitor treatment compliance might be inadequate.

In the present study, significant correlations were observed between the changes in axial length over 2 years and the age and SER at enrolment in the OK monotherapy group and showed larger increases in subjects with younger age and lower initial myopia. However, there were no significant correlations between the changes in axial length over 2 years and the age and SER in the combination therapy group (Figs. 3, 4). Therefore, we analysed the interactions between combination treatment and age and between combination treatment and SER for changes in axial length over 1 year or 2 years using a multiple linear regression analysis. These analyses revealed a tendency toward positive interaction between combination treatment and SER, with no significant interaction between combination treatment and age. When we stratified the subjects by SER at enrolment and then compared changes in axial length over 2 years, the difference in axial length increases between the two groups was found to be larger in subjects with a SER of − 1.00 to − 3.00 D than in all subjects with a SER of − 1.00 to − 6.00 D (Figs. 2, 5A). In contrast, there was no difference between the two groups in subjects with a SER of − 3.01 to − 6.00 D (Fig. 5B). These findings suggested that the suppressive effect of OK monotherapy on axial elongation may be affected by the initial SER of the children. The studies conducted by Kakita et al. and Hiraoka et al. have found that OK is less effective in slowing axial elongation in lower compared to higher degrees of myopia^20,21^. In their meta-analysis, Li et al. also reported that OK therapy alone slowed axial elongation more effectively in children with moderate to high initial myopia than with low initial myopia^22^. Therefore, the addition of 0.01% atropine to OK therapy may be more effective than OK monotherapy in slowing axial elongation in children with low initial myopia who had responded poorly to OK therapy, and as effective as OK monotherapy in children with moderate initial myopia who had responded strongly to OK therapy.

The anti-myopia mechanisms of OK and atropine remain unclear. OK is considered to slow the progression of myopia by an optical mechanism. As the myopia is increasingly corrected by OK, the progression may slow with the increase of higher order aberration due to a redistribution of the corneal epithelium^29,31^. Therefore, the effect of OK monotherapy in slowing the progression of myopia may be sufficiently strong in subjects with moderate initial myopia but not in subjects with low initial myopia. In contrast, because large pupil diameters facilitated the effect of OK to slow axial elongation in myopia^41^, atropine may suppress the progression of myopia by both pharmacological and optical mechanisms; anti-muscarinic and/or α 2-sympathomimetic effects, and an enhanced optical effect of OK by pupil enlargement. However, the pharmacological mechanism of atropine is likely not related to the amount of myopia correction. In the ATOM2 and LAMP studies, photopic pupil diameters in the 0.01% atropine group increased by 0.91 mm and 0.49 mm, respectively, 1 year after starting instillation compared to baseline^15,17^. Thus, it seems reasonable to assume that adding 0.01% atropine to OK therapy would be more effective in subjects with low initial myopia owing to the enhanced optical effect of OK by pupil enlargement. Because the effect of OK monotherapy in slowing axial elongation was sufficiently strong in subjects with moderate initial myopia, an enhanced optical effect of OK by pupil enlargement as the result of instillation of 0.01% atropine may not have been significant. The LAMP study demonstrated that low-concentration atropine administration reduced myopia progression with a concentration-dependent response^16,17^. Therefore, the combination of OK and higher concentration atropine, such as 0.025% or 0.05%, might make a significant difference, even in subjects with moderate initial myopia.

This study has some limitations. Although the pupil enlargement by atropine might have enhanced the effect of OK, we did not measure peripheral refraction, higher order aberration, or pupil diameter. These measures will need to be performed to clarify the mechanisms underlying the efficacy of combined OK and atropine therapy for slowing axial elongation in myopia. Second, we did not consider environmental factors, such as the amount of time subjects spent in near work or engaging in outdoor activities, and genetic factors such as the number of myopic parents. Third, we did not perform adjustments for multiple comparisons in the post-hoc analyses in this study to avoid confusing the readers’ interpretation. The results of the post-hoc analyses should be considered exploratory and the hypothesis based on the results obtained in this study must be revalidated in another study. Fourth, we excluded the subjects with poor compliance rates from the statistical analyses, because it was predicted in advance that the number of subjects with poor compliance rates in the combination therapy group would be larger compared to those in the OK monotherapy group. Statistical analyses including the subjects with poor compliance rates would be ideal. Fifth, we were not masked to the subjects. Originally, the administration of 0.01% atropine ophthalmic solution should be performed using a double-blind manner. Sixth, self-reporting by children and their parents or guardians as a method to monitor treatment compliance might be inadequate. Seventh, we did not understand why there was no difference between the two groups in the second year. This study could not provide evidence such as drug resistance to atropine. These issues should be considered in future studies.

During the 2-year follow-up period, the combination of OK and 0.01% atropine was more effective in slowing axial elongation than OK alone in children with myopia, especially in the first year. The efficacy of combined OK and 0.01% atropine therapy for slowing axial elongation was greater in children with low initial myopia. Combination therapy was equally as effective as OK monotherapy in children with moderate initial myopia because OK therapy slowed axial elongation more effectively in these children. Using 0.01% atropine ophthalmic solution together with OK therapy may be a usable and optimal treatment option to slow axial elongation, especially in children with low initial myopia.

## Supplementary information

Supplementary Information

## Data Availability

All data analysed during this study are included in this published article and its Supplementary Information files.
